# Development of a novel Cu (I) *π*‐complexation adsorbent for ultra‐deep desulfurization from a carbon dioxide stream

**DOI:** 10.1002/smo.20240027

**Published:** 2025-01-04

**Authors:** Huating Ju, Yongchun Zhang, Jikai Zhang, Ziqi Yu, Yige Zhang, Xiongfu Zhang, Xinwen Guo, Jiaxu Liu, Qing Mao, Qi Liu, Yiming Zhao, Tianqinji Qi, Xiao Jiang, Zhen Guo, Shaoyun Chen

**Affiliations:** ^1^ State Key Laboratory of Fine Chemistry Frontier Science Center for Smart Materials School of Chemical Engineering Dalian University of Technology Dalian China; ^2^ Engineering Building No.3 Hongo Campus The University of Tokyo Tokyo Japan; ^3^ Aramco Americas ‐ Boston Research Center Cambridge Massachusetts USA; ^4^ Cambridge Centre for Advanced Research and Education in Singapore Singapore Singapore

**Keywords:** *π*‐complexed adsorption, carbonyl sulfide, CO_2_ purification, Cu(I) adsorbent, desulfurization

## Abstract

Desulfurization technology is rather difficult and urgently needed for carbon dioxide (CO_2_) utilization in industry. A new Cu(I)‐based adsorbent was synthesized and examined for the capacity of removing carbonyl sulfide (COS) from a CO_2_ stream in an effort to solve the competitive adsorption between CO_2_ and COS and to seek opportunity to advance adsorption capacity. A wide range of characterization techniques were used to investigate the physicochemical properties of the synthesized Cu(I) adsorbent featuring *π*‐complexation and their correlations with the adsorption performance. Meanwhile, the first principal calculation software CP2K was used to develop an understanding of the adsorption mechanism, which can offer useful guidance for the adsorbent regeneration. The synthesized Cu(I) adsorbent, prepared by using copper citrate and citric acid on the ZSM‐5 (SiO_2_/Al_2_O_3_ = 25) carrier, outperformed other adsorbents with varying formulations and carriers in adsorption capacities. Through optimization of the preparation and adsorption conditions for various adsorbents, the breakthrough adsorption capacity (*Q*_*b*_) for COS was further enhanced from 2.19 mg/g to 15.36 mg/g. The formed stable *π*‐complex bonds between COS and Cu(I), as confirmed by density functional theory calculations, were verified by the significant improvement in the adsorption capacity after regeneration at 600°C. The above advantages render the novel synthesized Cu(I) adsorbent a promising candidate featuring cost‐effectiveness, high efficacy and good regenerability for desulfurization from a CO_2_ stream.

## Graphical abstract

The left side of this graphic illustrates the mechanism by which the newly synthesized Cu(I)‐based adsorbent absorbs carbonyl sulfide (COS). Cu atoms supported on ZSM‐5 can selectively absorb COS from a mixture gas stream flowing without adsorbing carbon dioxide. The S atom in COS can form a chemical bond, or alternatively, both the S and C atoms can simultaneously form chemical bonds with two adjacent Cu atoms. While the right side illustrates the increase in breakthrough capacity corresponding to each optimization step.

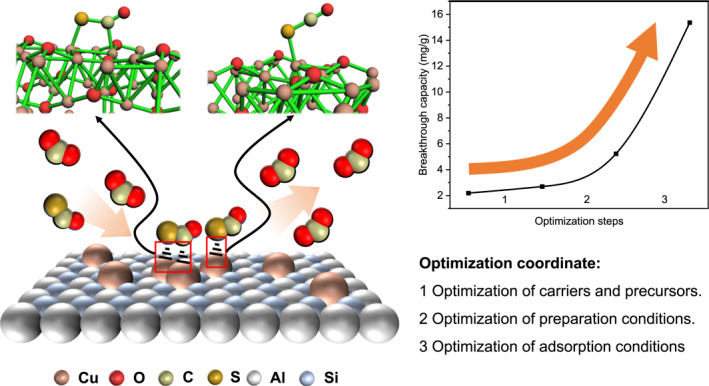

## 1 INTRODUCTION

As the major component of greenhouse gas, carbon dioxide (CO_2_) emissions are considered a primary contributor to the global climate change, which accounts for roughly 66% of the global warming caused by anthropogenic activities.^[1]^ The Paris Agreement was signed to commit to combat climate change through global efforts.^[2]^ To limit or even reduce the CO_2_ emissions, carbon capture, utilization, and storage (CCUS) technologies have been extensively studied and developed in the past decades to provide efficient and cost‐effective solutions.[^3^, ^4^] In the coal chemical industry, a large amount of CO_2_ is released along with gases impurities such as carbonyl sulfide (COS),^[5]^ hydrogen sulfide (H_2_S),^[6]^ ethylene (C_2_H_4_),^[7]^ etc, in the waste streams. After removing these impurities, the obtained CO_2_ can be used in various industries such as fertilizer for urea production and oil and gas industry for enhanced oil recovery, as well as in other commercial applications including food, electronics, medicine, etc.[^8^, ^9^] For some industries and applications, a strict control of the impurity concentrations has been applied. For example, the concentration of sulfide in food‐grade CO_2_ must not exceed 0.1 ppm.^[10]^ These mandatory criteria signify the need for highly selective and efficient purification technologies. COS is a major sulfide in impurities which not only poses significant threats to the environment and human health but also to equipment and pipelines and to catalyst performance. Health‐wise, exposure to COS at a concentration exceeding 40 mg/m^3^ can result in the damage to the nervous system, while the concentration above 240 mg/m^3^ is fatal.[^11^, ^12^] Industrial process‐wise, a long‐term exposure to COS will cause equipment corrosion and catalyst poisoning.^[13]^ In specific, the presence of O_2_ and H_2_O vapor in the streams can trigger the conversion of COS into H_2_SO_4_, which is corrosive to chemical equipment; sulfate and sulfur can form on the catalyst surface in the presence of COS, inevitably resulting in catalyst pore clogging and poisoning.^[14]^ Clearly, effective removal of COS from CO_2_ streams is essential to prevent these issues and to ensure the successful CO_2_ utilization in various industries.

Removing COS from CO_2_ streams presents significant challenges as each existing method has its own limitations. As shown in Table 1, the physical and chemical properties of COS and CO_2_ are similar, making the separation process difficult. Traditional physical adsorption methods have been extensively studied, with the adjustment of adsorbent pore size being a key factor in improving sorption capacity. Techniques such as alkaline treatment are commonly employed, while distillation and extraction are typically related to separating specific components.[^15^, ^16^] However, this method often presents ineffective separation.^[17]^ Other current prevailing methods for COS removal include absorption, hydrolysis, oxidation, reduction, and adsorption.^[18]^ Despite its wide application, absorption methods struggle to reduce COS concentrations below 0.1 ppm from CO_2_ streams.^[19]^ Oxidation and reduction methods are effective; however, they may generate undesired by‐products such as H_2_S and sulfur dioxide. Hydrolysis, another common approach, is frequently impeded by CO_2_ interference due to its competitive adsorption on the catalyst active sites.[^20^, ^21^] In contrast, chemical adsorption through *π*‐complexation has proven successful in reducing COS concentrations to below 0.1 ppm.^[19]^ For example, in our previous study, Wang et al. synthesized AgNO_3_‐modified NaZSM‐5 adsorbent, which exhibits great COS breakthrough adsorption capacity.^[22]^ However, the Ag(I) adsorbent has not been widely applied in industry due to its high price and being easily poisoned when exposed to H_2_S.^[23]^ Therefore, the present study continues our previous efforts in developing Ag(I)‐based adsorbents by addressing the challenges pertaining to developing cost‐effective alternative candidates for COS removal.

**TABLE 1:** Comparison of physical and chemical properties between carbonyl sulfide (COS) and carbon dioxide (CO_2_).

Physical and chemical properties	COS	CO_2_
Effective molecular diameter (nm)	0.36	0.33
Approximate acid‐base property of aqueous solution (pH)	6.5	5.6
Solubility (mL/100 mL H_2_O)	133.3	171.3
Boiling point (°C)	223.15	194.65

Exploring various materials with electronic structures similar to Ag(I) provide an avenue for seeking alternative candidates. Meanwhile, the preparation methods and strategies for enhancing stability for the new candidate(s) are essential. With an electronic configuration of 4d105s1, Ag's similar orbital structures in metal ions like Cu(I), Ni(II), and Zn(II) metal ions show similar orbital structures as Ag (electronic configuration, 4d^10^5s^1^), rendering them viable alternatives for replacing Ag(I).^[24]^ Besides, these metals are cost‐effective and less reactive with H_2_S than Ag(I) due to their lower tendency to form complexes or to undergo oxidation reactions with H_2_S.[^25^, ^26^] Different reducing agents (e.g., FeCl_2_,^[24]^ Na_2_SO_3_,^[27]^ VCl_3_,^[28]^ ammonium citrate^[29]^ and copper acetate (CAc)^[30]^) and different copper salts (e.g., CuCO_3_, CuCl_2_, CuSO_4_, and Cu(NO_3_)_2_ ^[24]^) have been reported to prepare Cu(I) adsorbent. CuCl was traditionally used as the precursor to prepare Cu(I)‐based adsorbent.[^28^, ^29^, ^30^] But CuCl is easy to be oxidized to Cu(II) by O_2_ during the preparation.[^31^, ^32^] To address this issue, alternative methods for adsorbent preparation have been proposed. Copper formate (CF) and CuCl_2_ were reported^[33]^ to prepare Cu(I), the calcination process of which generated hydrogen (H_2_) and carbon monoxide (CO), therefore reducing Cu(II) ions to Cu(I) ions.^[33]^ However, the CF‐ and CuCl_2_‐derived adsorbents did not exhibit competitive performance,^[33]^ likely due to the limited reduction capacity of these agents in achieving effective Cu(I) formation. Likely, switching to different reducing agents provides a way. Considering its structural similarity to CF, copper citrate (CC) is a potential alternative to prepare high‐performance Cu(I) adsorbent. In addition to identifying the appropriate reducing agents, efforts in further improvements focus on enhancing the stability of the adsorbent. Sun et al. have signified that Cu ^+^ cannot be oxidized by oxygen when no moisture presents, and the hydrophilia of Cu(I) surface can be modified with polydimethylsiloxane coating.^[34]^ Moreover, Wang et al. have suggested that the carriers with pore sizes close to COS Effective Molecular Diameter prefer to adsorb COS.^[22]^ While these experimental optimizations have led to significant advancement in stability and adsorption performance, theoretical studies have also provided insights into further improvements by unveiling the crucial molecular interactions during the adsorption. Pavan et al. have employed the CP2K and Multiwfn software to characterize the bond lengths, energy changes, and orbital bonding of molecules in the adsorption,[^32^, ^33^, ^35^] shedding light on understanding the experimental results.

The objective of the present work is to develop a novel, highly efficient, and cost‐effective COS adsorbent that offers a breakthrough in performance. By beginning, various metal ion‐based adsorbents had been tested for COS adsorption with Ag(I)‐based adsorbent serving as the benchmark. Then, the relationship of adsorption capacities of selected metal‐ion adsorbents and effects of reducing agents and precursors has been studied, followed by exploring the influence of carriers. To further optimize the adsorption capacity, tests were carried out under different adsorption conditions. Nitrogen (N_2_) atmosphere had been employed throughout all tests to maintain an anhydrous condition. Theoretical adsorption models of COS and CO_2_ adsorbed on different adsorbent surfaces (Cu_2_O, NiO, CuO, and ZnO) were developed using density functional theory (DFT) simulations, the results of which were associated with phenomenological observations. From the practical perspective, simulation results suggest suitable temperatures for the adsorbent regeneration, which was later validated by experiments, offering a novel approach to provide insights into the reusability and effectiveness of newly developed adsorbents.

## 2 RESULTS AND DISCUSSION

### 2.1 Screen of the transition metal ions

**FIGURE 1:**
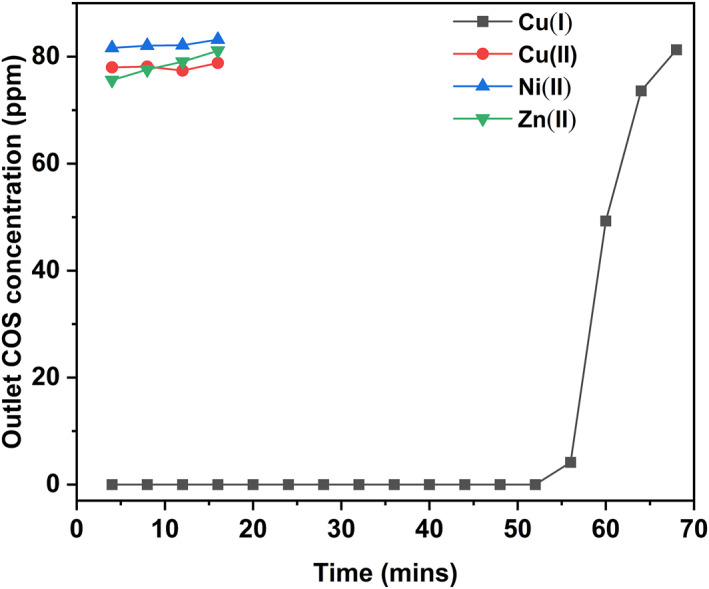
Breakthrough curves of Cu(I), Cu(II), Ni(II), and Zn(II) modified adsorbents. Adsorption condition: 70°C, carbonyl sulfide/carbon dioxide gas flow rate of 50 mL/min, and adsorbent dosage of 0.3 g.

Figure 1 shows the COS breakthrough curves of four selected transition‐metal‐ions‐based ZSM‐5 adsorbents. For a duration of 52 min, COS was not detected at the outlet using Cu(I)‐based adsorbent. In contrast, the COS outlet concentration was maintained at about 80 ppm using Cu(II)‐based, Ni(II)‐based, and Zn(II)‐based adsorbents. This indicates that only Cu(I) can successfully remove COS, and it has the potential to be used for the development of efficient desulfurization adsorbents. The other ions did not show the same desulfurization effect as Cu(I). The main reason may be attributed to the weak *π*‐complexation capacity of Cu(II), Ni(II), and Zn(II) compared to Cu(I).^[23]^ So, it is of interest to explore appropriate methods for preparing stable Cu(I) adsorbents in the next step.

### 2.2 Characterization of the Cu(I) adsorbents

**FIGURE 2:**
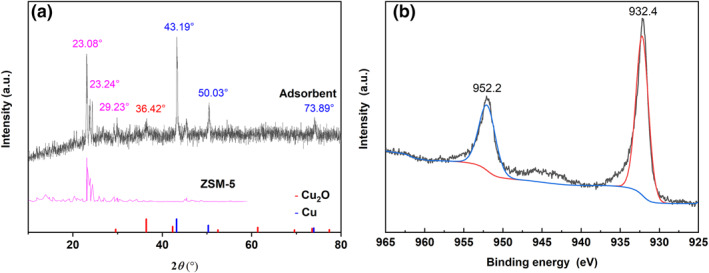
(a) X‐ray diffraction patterns and (b) X‐ray photoelectron spectroscopy spectrum of the as‐prepared Cu(I)/ZSM‐5 adsorbent.

X‐ray diffraction (XRD) patterns of the Cu(I)/ZSM‐5 adsorbent are shown in Figure 2a. The diffraction peaks at 23.08°, 23.24°, and 29.23° can be indexed to respective (501), (051), and (352) planes of the ZSM‐5 carrier by using Jade 6.5 software. 2*θ* = 43.19°, 50.30°, and 73.89° are the diffractions of Cu phase and can be indexed to the (111), (200), and (220) planes, respectively. 2*θ* = 36.42° is the diffraction peak of Cu_2_O phase and corresponds to the (111) plane. The surface property of the adsorbent was investigated by X‐ray photoelectron spectroscopy (XPS). Figure 2b shows the XPS spectrum of the Cu(I)/ZSM‐5 adsorbent. The peaks at 932.4 and 952.2 eV are assigned to Cu2p^3/2^ and Cu2p^1/2^ of Cu(I).^[36]^ Corresponding peaks of Cu(II) do not appear at 934.7 and 954.7 eV.^[29]^ Clearly, these bulky and surface characterization results indicate the presence of Cu(I) in the form of Cu_2_O and the absence of Cu(II).

**FIGURE 3:**
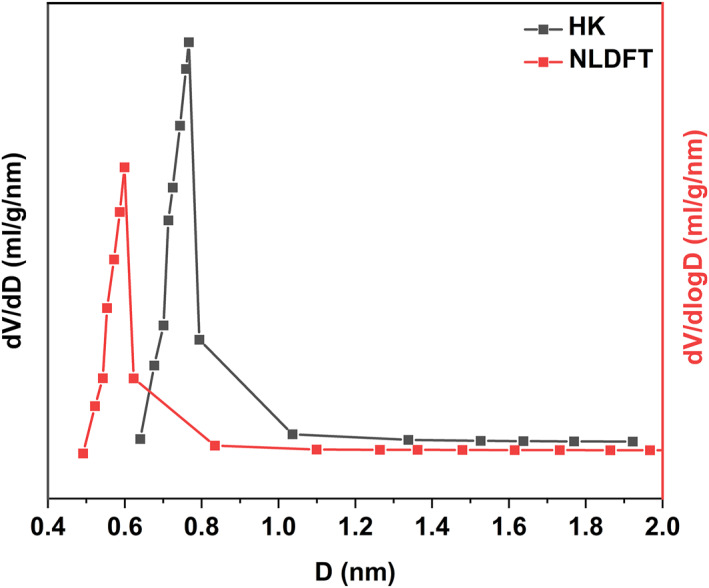
Pore size distribution (*d*_*P*_) of the Cu(I)/ZSM‐5 adsorbent using (a) HK method and (b) NLDFT method. HK, Horvath‐Kawazoe; NLDFT, non‐local density functional theory.

Figure 3 presents the pore size distribution (*d*_*P*_) of the Cu(I)/ZSM‐5 adsorbent, and the results were calculated using N_2_‐physisorption isotherms and processed by both Horvath‐Kawazoe (HK) and Non‐Local Density Functional Theory (NLDFT) methods. Using the HK method, *d*_*P*_ was concentrated in the range of 0.6−1 nm, while it was concentrated in the range of 0.5−0.9 nm using the NLDFT method. Both results show that the *d*_*P*_ range of the adsorbent is slightly larger than the COS effective molecular diameter, which is suitable for the removal of COS.^[22]^

**FIGURE 4:**
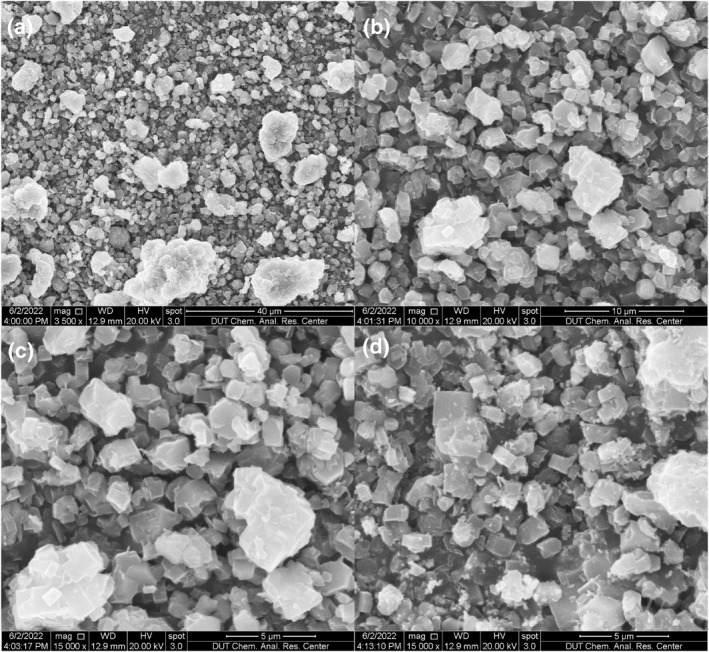
SEM (a and b) and transmission electron microscope (c and d) images of Cu(I)ZSM‐5 adsorbents.

Figure 4 displays the SEM and Transmission electron microscope images of Cu(I)/ZSM‐5 adsorbents. In Figure 4a,b, Cu(I)/ZSM‐5 presents the characteristic hexahedral structure of ZSM‐5 at the magnification of 15,000 times. In Figure 4c,d, adsorbents are hexahedrons with a diameter of ca. 1 μm. Clearly, the hexagonal structure of ZSM‐5 was well‐retained post the adsorbent preparation. In other words, loading active Cu(I) species onto ZSM‐5 matrix barely changes the matrix of the carrier.

### 2.3 Screening of carriers and precursors

**FIGURE 5:**
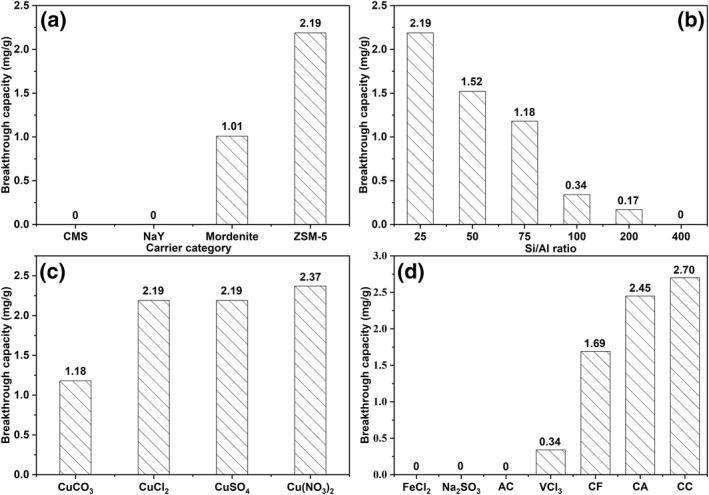
Breakthrough capacities (*Q*_*b*_) of adsorbents using different (a) carriers, (b) SiO_2_/Al_2_O_3_ ratios of ZSM‐5, (c) copper salts, and (d) reducing agents. Adsorption conditions: 70°C, carbonyl sulfide/carbon dioxide gas flow rate 50 mL/min, adsorbent dosage 0.3 g.

With the selected alternative Cu(I) ions, important factors of carriers on adsorption performance were investigated, including SiO_2_/Al_2_O_3_ ratios, copper salts, and reducing agents. Due to the similarity between the pore size and the kinetic diameter of COS, the following zeolite materials, Carbon Molecular Sieve (CMS), Sodium Y Zeolite (NaY), Mordenite (MOR), and ZSM‐5, have been selectively considered. The comparisons between different variables are presented in Figure 5.

Figure 5a shows the *Q*_*b*_ of adsorbents featured with varying carriers. Both CMS and NaY as carriers show negligible COS adsorption capacities. The COS *Q*_*b*_ of ZSM‐5 (2.19 mg/g) exhibited 1.2 times higher adsorption capacity than that of MOR (1.01 mg/g). Clearly, ZSM‐5 is a more suitable carrier for COS removal among the carriers examined.

Figure 5b displays the COS *Q*_*b*_ of Cu(I)/ZSM‐5 adsorbents with varying SiO_2_/Al_2_O_3_ ratios. The *Q*_*b*_ for Cu(I)/ZSM‐5 decreases significantly as the SiO_2_/Al_2_O_3_ ratio increases and even presents negligible adsorption performance when the ratio is as high as 400. Among all samples, ZSM‐5(25), with SiO_2_/Al_2_O_3_ ratio of 25, has the highest *Q*_*b*_ of 2.19 mg/g.

Figure 5c shows the COS *Q*_*b*_ capacity for adsorbents with different copper salts. CuCO_3_‐derived adsorbent displays the lowest *Q*_*b*_ of 1.18 mg/g. COS *Q*_*b*_ of CuCl_2_‐and CuSO_4_‐derived adsorbents show similar adsorption performance at 2.19 mg/g. The Cu(NO_3_)_2_ adsorbent exhibits the highest *Q*_*b*_ at 2.37 mg/g. Further studies use Cu(NO_3_)_2_ as copper salt.

Figure 5d shows the COS *Q*_*b*_ for adsorbents with varying reducing agents. Using FeCl_2_ and Na_2_SO_3_ as reduced components is ineffective for removing COS (0 mg/g). The Q_b_s of VCl_3_, CF, CA are 0.34, 1.69, and 2.45 mg/g, respectively. The formulation of CC and citric acid (CA) had the highest *Q*_*b*_ of 2.70 mg/g. For CA's case, the highest adsorption capacity is likely because of the paired one acid root with one Cu(II). In specific, the CA molecule is composed of two acetate roots and one copper ion, so additional copper salts need to be added to ensure that the ratio of acid to copper ion is 1:1. While the CC molecule is composed of one citrate root with two copper ions, CA needs to be added to ensure that the ratio of acid to copper ion is 1:1.^[31]^ Further studies will use CC and CA as the precursors for preparing adsorbents.

### 2.4 Effect of preparation conditions

Post the optimization of preparation materials and precursors, adsorption tests were conducted under varying conditions to seek opportunities to further optimize the adsorption capacity. Parameters that had been examined included temperatures, contact time, adsorbent dosage amount, and gas flow rate.

**FIGURE 6:**
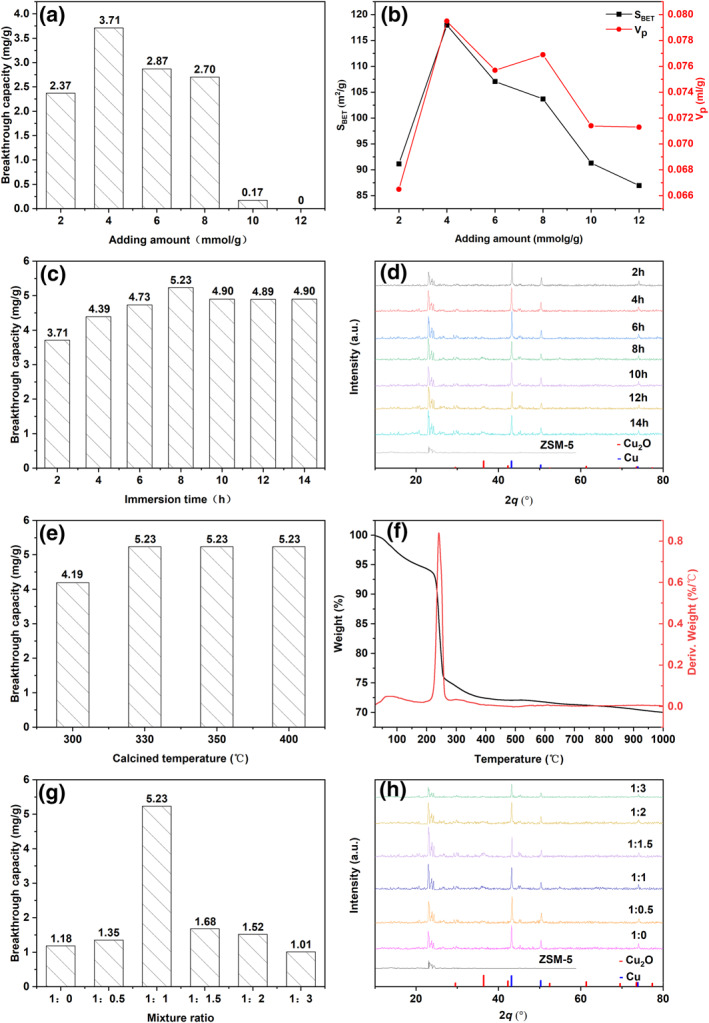
(a) Breakthrough capacities of adsorbents with different Cu amounts. (b) *S*_BET_ and *V*_*P*_ of adsorbents with Cu amounts. (c) Breakthrough capacities. (d) X‐ray diffraction (XRD) patterns of adsorbents with different immersion times. (e) Breakthrough capacities of adsorbents with calcination temperatures. (f) Thermogravimetry differential thermal analysis curve of the adsorbent. (g) Breakthrough capacities and (h) XRD patterns of adsorbents with different copper citrate/citric acid mixing ratios.

Figure 6a shows the COS *Q*_*b*_ of the adsorbents with different amounts of copper, which were prepared using the following conditions: 2‐h immersion, 330°C calcination temperature, and 1:1 mixing ratio of CC/CA. The *Q*_*b*_ initially increases with the increase in copper amount, followed by decreasing after reaching the maximum capacity at 3.71 mg/g with 4 mmol/g of Cu loading.

The Brunauer‐Emmett‐Teller (BET) surface area (*S*_BET_) and pore volume (*V*_*P*_) of adsorbents with different Cu amounts are compared in Figure 6b. Both *S*_BET_ and *V*_*P*_ increase with the increasing Cu amount. Above 4 mmol/g, the *S*_BET_ begins to decrease. These trends in *S*_BET_ and *V*_*P*_ are consistent with that of the Cu amount‐dependent COS *Q*_*b*_ in Figure 6a, indicating the correlations between *S*_BET_, *V*_*p*_, and *Q*_*b*_. In specific, an increase in *S*_BET_ and *V*_*P*_ leads to an improved *Q*_*b*_.

The immersion time for preparing the adsorbent is typically set to a minimum of 2 h. However, the duration is gradually increased until the point where further increase does not give any significant change in the adsorption capacity. Figure 6c shows the COS *Q*_*b*_ for adsorbents with different immersion times using the following preparation conditions: 4 mmol/g Cu amount, 330°C calcination temperature, and 1:1 mixing ratio of CC/CA. The *Q*_*b*_ increases significantly with the increasing immersion time and then decreases slightly after the optimal immersion time. The optimal immersion time is 8 h, and the highest *Q*_*b*_ is 5.23 mg/g. Figure 6d shows the XRD patterns of the adsorbents with immersion times. At 2*θ* = 36.42°, the peak area of Cu_2_O increases with the increasing immersion time and then barely changes or decreases slightly beyond 8‐h immersion time, which is consistent with the change in the immersion‐time‐dependent *Q*_*b*_. Figure 6c,d imply that more Cu_2_O phases are loaded with the increasing immersion time shorter than 8 h. When the immersion time exceeds 8 h, increasing the immersion time can no longer increase the *Q*_*b*_.

Figure 6e shows the COS *Q*_*b*_ of adsorbents with calcination temperatures with the following preparation conditions: 4 mmol/g Cu amount, 8‐h immersion time, and 1:1 mixing ratio of CC/CA. The *Q*_*b*_ increased as the temperature increased and then became stable beyond 330°C. Figure 6f shows the Thermogravimetry Differential Thermal Analysis curves of the adsorbent. The TG curve decreased significantly and then became stable beyond 300°C with a weight loss of 26%. The DTA curve shows an exothermic peak at 240°C, which is due to the decomposition of citrate during calcination. The decomposition of citrate likely releases H_2_ and CO, which could reduce the Cu(II) to Cu(I). The DTA curve suggests that the calcination temperature should be at least 300°C to decompose the citrate completely, below which it is not conducive to the generation of Cu(I). This is consistent with the study in Figure 6e.

Figure 6g shows the COS *Q*_*b*_ for adsorbents with CC/CAc mixing ratios with the following preparation conditions: 4 mmol/g Cu amount, 8‐h immersion time, and 330°C calcination temperature. The *Q*_*b*_ exhibits a volcano shape with the increase in the CC/CA ratio. The highest *Q*_*b*_ of 5.23 mg/g was obtained at a CC/CAc ratio of 1:1. Figure 6h presents the XRD patterns of the adsorbents with different copper/acid ratios. The Cu_2_O diffraction peak was at 36.42°. As the CC/CA ratio approaches 1:1, the intensity of Cu_2_O diffraction peak maximizes.

### 2.5 Effect of adsorption conditions

To further optimize the adsorption performance, effects of adsorption parameters were investigated, including temperature, gas compositions, and contact time. During the tests, the flow rate varies from 50 to 110 mL/min, and the adsorption temperature is from 100 to 400°C.

**FIGURE 7:**
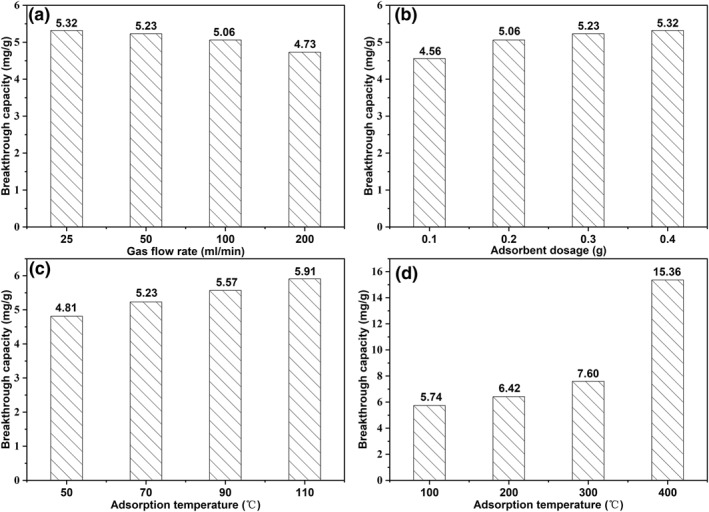
Breakthrough capacities of adsorbents with different (a) gas flow rates, (b) adsorbent dosages, and (c and d) adsorption temperatures.

The COS *Q*_*b*_ of 0.3 g adsorbents with different gas‐flow rates at 70°C is shown in Figure 7a. The COS *Q*_*b*_ exhibits a slight decrease with the increase in flow rates, which can be ascribed to the shortened contact time caused by high flow rate. Figure 7b shows the COS Qb of adsorbents with varying dosages of active metal ion components, while the total mass of the adsorbent, including the carrier, remains constant. The results were collected at 70°C and 50 mL/min COS/CO_2_ gas flow rate. The *Q*_*b*_ increases with an increase in dosage, which might be due to the prolonged contact time during the adsorption. Figure 7c,d show the COS *Q*_*b*_ of adsorbents at different adsorption temperatures, wherein (c) is between 50 and 110°C, and (d) is at higher temperatures (e.g., 100–400°C). Adsorption conditions were 50 mL/min COS/CO_2_ gas flow rate and 0.3 g adsorbent dosage. In these two figures, the *Q*_*b*_ increases as the adsorption temperature increases. Such a trend indicates that the increase in temperature favors the *π*‐complexed adsorption of COS. Likely, the adsorbent requires high temperatures to overcome intermolecular forces to form *π*‐complexed bonds with COS.

As shown in Table 2, the sensitivity analysis of key preparation parameters for COS adsorbents reveals several important correlations. The SiO_2_/Al_2_O_3_ ratio has a strong effect on the performance with a ratio of 25 yielding the highest *Q*_*b*_ value before decreasing at higher ratios. Among the copper salts tested, Cu(NO_3_)_2_ achieved the best result. For reducing agents, the CC/CA mixture provided the highest *Q*_*b*_ value with an optimal mixing ratio of 1:1. The amount of copper added also played a crucial role with 4 mmol/g being the most effective amount. Soaking the material for 8 h yielded the best adsorption results, and calcination at 330°C was found to stabilize *Q*_*b*_. These insights offer clear directions for optimizing the preparation of high‐performance COS adsorbents.

**TABLE 2:** Summary of sensitivity analysis for key parameters that affect *Q*_*b*_ during carbonyl sulfide (COS) adsorption.

Parameter	Range	Key findings	Optimal condition
SiO_2_/Al_2_O_3_ ratio	25–400	*Q*_*b*_ reaches its max. peak at a ratio of 25, then decreases	25
Copper salt type	CuCO_3_, CuCl_2_, CuSO_4_, Cu(NO_3_)_2_	Cu(NO_3_)_2_ exhibits the highest *Q*_*b*_ value	Cu(NO_3_)_2_
Reducing agent type	FeCl_2_, Na_2_SO_3_, VCl_3_, CF, CAc, CC/CA	CC/CA mixture shows the highest *Q*_*b*_ value	CC/CA
Copper addition amount	2–6 mmol/g	*Q*_*b*_ is highest at 4 mmol/g, decreasing afterward	4 mmol/g
Soaking time	2–10 h	Soaking time of 8 h results in the highest *Q*_*b*_	8 h
Calcination temperature	300–400°C	*Q*_*b*_ stabilizes at 330°C	330°C
CC/CA mixture ratio	1:0.5–1:2	*Q*_*b*_ peaks at a CC/CA ratio of 1:1, higher ratios decrease *Q*_*b*_	1:1

### 2.6 Theoretical simulation for COS adsorption mechanism

**FIGURE 8:**
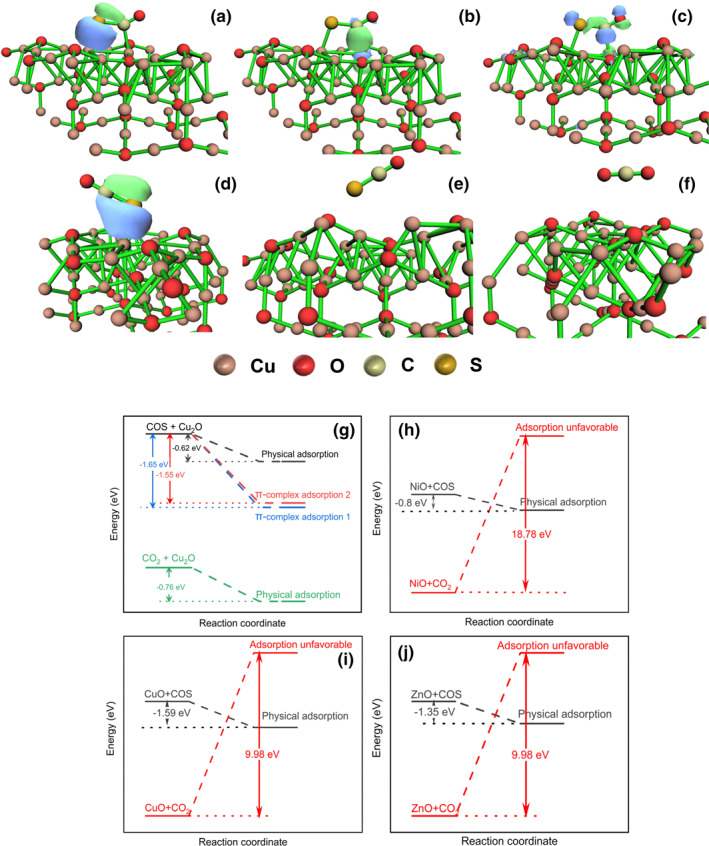
*π*‐Complexed adsorption of carbonyl sulfide (COS) on Cu_2_O with (a–c) Type 1 and (d) Type 2. (e and f) Physical adsorption of COS and carbon dioxide (CO_2_) on Cu_2_O. (g) Gibbs free energy profiles for the COS and CO_2_ adsorption on Cu_2_O (from CP2K calculations, level of theory: PBE‐D3; basis set: DZVP‐MOLOPT‐SR‐GTH), (h) NiO (level of theory: revPBE‐D3; basis set: DZVP‐MOLOPT‐SR‐GTH), (i) CuO, and (j) ZnO.

Figure 8 shows the localized molecular orbital (LMO) analysis of the COS *π*‐complexed adsorption on Cu_2_O. Two types of *π*‐complexed adsorption were identified. Figure 8a–c show the molecular orbitals for Type 1 adsorption, where both the carbon and sulfur atoms in the COS molecules are bonded to the Cu₂O slab, with C‐Cu and S‐Cu bond lengths of 2.05 and 2.17 Å, respectively. In contrast, Figure 8d presents the molecular orbitals for Type 2 adsorption, where only sulfur in the COS molecule bonds with the Cu₂O slab, with an S‐Cu bond length of 2.21 Å. Figure 8a,d demonstrate that the carbon and sulfur atoms bonded with Cu on Cu_2_O slab surface. The localized orbital occupation shows that the *π*‐complexed adsorption occurs. The bond lengths of S‐Cu in Type 1 adsorption are shorter than those of Type 2 adsorption, indicating that these bonds in Type 1 adsorption are more stable than those in Type 2 adsorption. Additionally, compared to free molecules, there are some structural variations for adsorbed COS molecules. The length of the C‐S bond is elongated from 1.56 to 1.68 Å in Type 1 adsorption and from 1.56 to 1.59 Å in Type 2 adsorption, indicating that the strength of the C‐S bond is decreased, and COS is activated on the surface of the adsorbent.

Figure 8e shows that COS is adsorbed through van der Waals forces, indicating physisorption. Similarly, Figure 8f demonstrates that CO₂ adsorption on the Cu(I) surface is also physical adsorption, with no chemical or *π*‐complexed interactions observed. This indicates a more selective COS adsorption over CO_2_ on the adsorbents through the *π*‐complexation. In physisorption, the length of the S‐Cu bond between COS and Cu_2_O is 3.06 Å, while the distance between CO_2_ and Cu_2_O is 3.46 Å. The bond lengths of physical adsorption are longer than those of *π*‐complexed adsorption, indicating that *π*‐complexed adsorption is more stable.

The Gibbs free energy profiles of COS and CO_2_ adsorption on Cu_2_O, NiO, CuO, and ZnO are illustrated in Figure 8g. The energy barriers referenced to COS and CO_2_ adsorbed on Cu(I) adsorbents are −0.62 eV and −0.76 eV, respectively. The chemical COS adsorption barriers of type 1 and type 2 *π*‐complexed adsorption are −1.65 eV and −1.55 eV, respectively, which are lower than the physical adsorption barriers. The adsorption energy barriers for COS and CO₂ on NiO are −0.8 and +18.78 eV, respectively, indicating that the COS on Ni(II) is physical adsorption (Figure 8h). CO_2_ is adsorption unfavorable with the positive adsorption energy. Figure 8i,j show the adsorption energies (respective −1.59 and −1.35 ev) of COS adsorbed on Cu(II) and Zn(II) surface, the *π*‐complexed adsorption does not form from the DFT calculation. The adsorption of COS on Zn(II) is more likely the interaction between S and O of the ZnO surface. The result of the simulation of adsorption is in line with the experimental findings in performance trends.

Based on the identified different types of COS adsorption on Cu surfaces and their associated molecular orbitals and energy analyses presented in Figure 8, the adsorption and desorption mechanisms of COS on Cu(I) are shown as follows:

Adsorption:

(1) Cu+COS→Cu–SCO

(2) 2Cu+COS→Cu–CO–Cu–S

The adsorption can be described through two primary mechanisms. In the first mechanism, COS adsorbs onto the Cu surface via the sulfur atom (Cu + COS → Cu − SCO). This interaction results in a direct chemical bond between the sulfur atom of COS and the Cu surface, forming a stable adsorption structure. In the second mechanism, both carbon and sulfur atoms of COS participate, leading to a bridge‐like adsorption structure where COS is coordinated between two Cu atoms (2Cu + COS → Cu − CO − Cu − S). This bridge structure demonstrates the ability of the Cu surface to accommodate multiple points of contact with the COS molecule, enhancing the stability of the adsorption.

Desorption:

(3) Cu−COS→Cu+COS

(4) Cu−CO−Cu−S→Cu+COS

The desorption of COS can occur via two distinct mechanisms as well. In the first mechanism, COS is released from the Cu surface through the breaking of the Cu−SCO bond (Cu − COS → Cu + COS), returning to its original state. In the second mechanism, the breakdown of the Cu − CO − Cu − S bridge structure facilitates the release of COS (Cu − CO − Cu − S → Cu + COS), indicating that even the bridge structure, despite stable, can be dissociated under certain conditions.

### 2.7 The regeneration of the adsorbents

**FIGURE 9:**
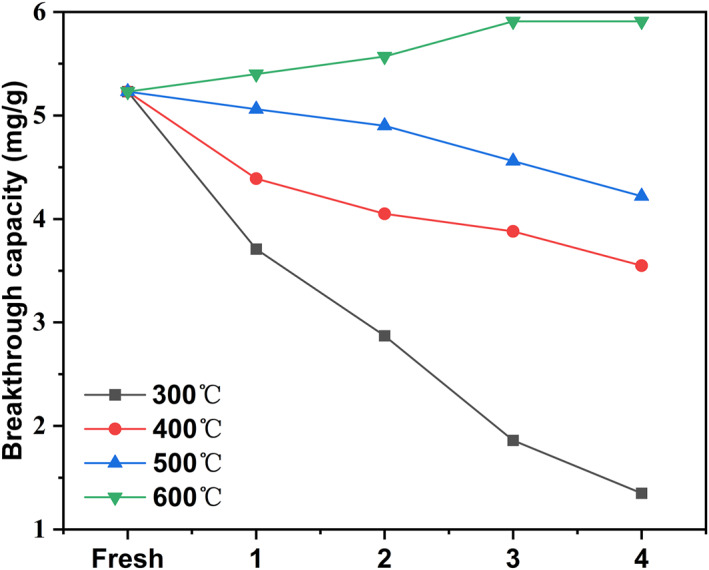
Regeneration capacities of Cu(I)/ZSM‐5 adsorbents at regeneration temperatures.

With the analysis of the bonding of the COS *π*‐complexed adsorption on Cu_2_O, the adsorbent and desorption require higher temperatures to form or rupture *π*‐complexed bonds with COS. The regeneration was conducted in a N_2_ stream at different temperatures without the addition of an aid desorption activator. Figure 9 shows the regeneration capacities of adsorbents at temperatures. The regeneration capacity increases with increasing regeneration temperatures during multiple adsorption‐regeneration cycles.

As revealed by simulations, two types of adsorptions, namely *π*‐complexed chemisorption and physisorption, occur between Cu_2_O and COS. Physisorption is weak, so the COS desorption on Cu_2_O does not require an extremely high temperature. The adsorbents can thus be partially regenerated at 300−500°C. The *π*‐complexed chemisorption is stronger and is more stable than physisorption. In this case, COS desorption requires higher temperature treatments, and the adsorbent can be completely regenerated at 600°C. At a regeneration temperature of 600°C, the *Q*_*b*_ exhibits a gradual increase with each regeneration cycle, before eventually reaching a stable value. Table 3 shows the comparison of S_BET_ and *V*_*P*_ between the fresh and regenerated adsorbents after cycles at 600°C. Noticeably, *V*_*P*_ barely changes, while S_BET_ increases significantly from 171.79 m^2^/g to 194.97 m^2^/g. The high‐temperature calcination‐induced increases S_BET_ likely correlate with the corresponding increases in *Q*_*b*_.

**TABLE 3:** *S*_BET_ and *V*_*P*_ of Cu(I)/ZSM‐5 adsorbents before and after 600°C regeneration.

Adsorbent status	*S*_BET_ (m^2^/g)	*V*_*p*_ (mL/g)
Fresh adsorbent	171.79	0.1109
After regeneration	194.97	0.1087

## 3 CONCLUSION

In this study, a novel Cu(I)‐based desulfurization adsorbent for removing COS from a CO_2_ stream was developed. Compared to conventional COS removal technologies and other ZnO, CuO, and NiO‐based adsorptive materials, the developed Cu(I)‐based adsorbent in this study, prepared by a mixture of CC and CA and loaded on ZSM‐5(25) carriers, shows significant advantages in COS removal. After optimization, the *Q*_*b*_ of Cu(I)/ZSM‐5 reached as high as 15.36 mg/g, outperforming other examined adsorbents under the same testing conditions. The optimized key factors included Cu amount (4 mmol/g), immersion time of 8 h, calcinature temperature of 330°C, adsorption temperature of 400°C, and a CC/CA mixing ratio of 1:1. DFT calculations reveal that ZnO, CuO, and NiO merely show weak bondings with COS via physisorption, while Cu_2_O presents stronger bonding with COS through chemisorption. The COS *Q*_*b*_ of adsorbent exhibits a gradual increase in the first three cycle regeneration tests and eventually reaches a stable value beyond three times with the regeneration temperature at 600°C. The synthesized Cu(I) adsorbent demonstrated improved adsorption performance in experimental scale but need further scale‐up validation on the pursuit of industrial applications.

## 4 EXPERIMENTAL METHODS

### 4.1 Materials

The source and purity of raw materials are shown in Table 4.

**TABLE 4:** Materials, sources, and purities used in experiments.

Material name	Source	Purity
ZSM‐5 (SiO_2_/Al_2_O_3_ 25−800)	Zhuoran Environmental Protection (Dalian) Co., Ltd.	/
MOR	Powder as self‐provided	/
NaY		/
CMS		/
CA	Shanghai Aladdin Biochemical Technology Co., Ltd.	≥99.5%
CC		99.5%
AC		98.5%
VCl_3_		97%
CF	Shanghai Macklin Biochemical Co., Ltd.	98%
FeCl_2_		98%
CuCl_2_	Tianjin Damao Chemical Reagent Factory.	99%
CuCO_3_ (measured by Cu)		52.5−56.5%
Na_2_SO_3_		≥97%
Ni(NO_3_)_2_		98%
CA	Tianjin Guangfu Chemical Reagent Factory.	≥99%
Cu(NO_3_)_2_	Sinopharm Chemical Reagent Co., Ltd.	≥99%
Zn(NO_3_)_2_		≥99%
CuSO_4_	Tianjin University Chemical Reagent Factory, Dongli District, Tianjin.	99%
100 ppm COS/CO_2_	Dalian GuangMing Special Gas Products Co., Ltd.	/
N_2_		99.999%

### 4.2 Preparation of adsorbents

In order to compare the *Q*_*b*_ under different preparation and adsorption conditions, it is necessary to establish a standard procedure for synthesizing absorbents. For this purpose, each absorbent was prepared by 2.0 g carriers, 2.0 mmol reducing agents, 2.0 mmol copper salt, and 100 mL deionized (DI) water. The mixture was then placed into a beaker and mixed using magnetic stirring at 1000 rpm for 30 min to form a suspension at room temperature and atmospheric pressure. The suspension was macerated for 2 h and then dried at 100°C for 12 h. The dry samples were crushed and sieved to 40−60 mesh and loaded into U‐type tubes. The samples were calcined at temperatures in N_2_ atmosphere and then cooled naturally to room temperature before adsorption tests.

### 4.3 Characterization of the adsorbents

Powder XRD (D/MAX‐2400) patterns were obtained from a Rigaku diffraction meter operated at 45 kV and 200 mA using Ni‐filtered Cu Kα radiation at a rate of 10°/min from 2*θ* = 10°–80°. N_2_ physisorption was performed at −196°C using a BSD‐PS2 to measure BET surface area (*S*_*BET*_), pore volume (*V*_*p*_), and pore size distribution (*d*_*p*_). All samples were dehydrated at 200°C for 2 h prior to the N_2_ adsorption. The XPS was conducted by using an ESCALAB XI + X‐ray photoelectron spectrometer. XPS data were calibrated using C1s as a reference with the binding energy at 284.6 eV. The thermal gravimetric/differential thermal analysis (TGA/DTA) was taken by SDTQ600 TA. Scanning electron microscope (SEM) images were taken using QUANTA 450. Transmission electron microscope (TEM) images were taken using Tecnai G220 S‐twin. CP2K was used to simulate theoretical models. A multifunctional wavefunction analyzer (Multiwfn) was used to analyze molecular orbitals.

### 4.4 Adsorption procedure

A gas stream of 100 ppm COS/CO_2_ was introduced into a U‐type tube, and the outlet gas concentration was monitored by a gas chromatograph equipped with a Flame Photometric Detector. The breakthrough concentration of COS was set at 0.1 ppm. The equation to calculate the *Q*_*b*_ of the adsorbents is shown as follows.

$$C_{\text{ap}}=\frac{T\times Q\times C_{\mathrm{COS}}\times M_{\mathrm{COS}}}{V_{\text{mol}}\times m}$$

*C*_ap_: *Q*_*b*_ of adsorbent (mg/g); *T*: Breakthrough time (min); *Q*: Gas flow rate (mL/min); *C*_COS_: The concentration of COS in the COS/CO_2_ gas, this experiment is 100 ppm; *V*_mol_: Molar volume at room temperature (15°C in winter) at atmospheric pressure, 23.7 (L/mol); *m*: Dosage of adsorbent (g); *M*_COS_: The molar weight of COS, 60.017 (g/mol).

### 4.5 Simulation method

The open‐source software CP2k^[37]^ was used to optimize the structure and to perform frequency calculations for NiO, CuO, Cu_2_O, and ZnO adsorbents. At first, Cu_2_O, CuO, and ZnO bulk cells were calculated, followed by cleaving the surface and building a 15‐A vacuum slab along the *Z* direction to avoid the interaction of adjacent cells. The atom orbitals were described by DZVP‐MOLOPT, in which the truncation energy of the plane and the relative truncation energy were considered by 400 Ry and 55 Ry. The electron is described by Goedecker Teter Hutter pseudopotential. The electronic structure is described by the Perdew Burke Ernzerhof functional under the generalized gradient approximation. The Gamma (Γ) Centered 7 × 7 × 7 k‐point is applied to the Brillouin zone for the Cu_2_O, CuO, and ZnO primitive cells, and the built slab and supercell are considered only the gamma Point. The van der Waals interaction is described by Grimme D3 dispersion correction. The Gibbs free energies of adsorption were corrected using Shermo software.^[38]^ While the NiO cell exhibits antiferromagnet, the magnetic moment of Ni has been defined as 2 and ‐2 for different layers of atoms. The orbital transformation method has been used to calculate the structural optimization and frequency calculations. The molecular orbital analysis of the results used different methods: LMOs, the partial density of states (PDOS), and overlap PDOS (OPDOS) by the Multiwfn software.^[39]^

## CONFLICT OF INTEREST STATEMENT

The authors declare no conflicts of interest.

## ETHICS STATEMENT

No animal or human experiments were involved in this study.

## Data Availability

Data are available on request from the authors.
